# Four Representations of the Skull of the Simia Satyrus, at Different Periods of Life, with the View of Illustrating the Fable of the Orang Utang

**Published:** 1840-07

**Authors:** 


ART. XII.—*Vier Abbildungen des Schädels der Simia Satyrus, von verschiedenen Alter, zur Erklärung der Fabel vom Oran Utan.* Herausgegeben von Dr. C. F. HEUSINGER.—*Marburg*, 1838.

*Four Representations of the Skull of the Simia Satyrus, at different Periods of Life, with the view of illustrating the Fable of the Orang Utang.* By Dr. C. F. HEUSINGER.—*Marburg*, 1838. 4to, pp. 44.

THE author purchased, in 1828, from Dr. Besel, who brought them with him from Batavia, besides the skin and complete skeleton, the skulls of three young orang utangs, which he deposited in the museum of the Zootomical Institute, founded by himself, of the university of Würzburg. In addition, he obtained from the same traveller a drawing of the cranium of an old orang, or pongo, which had been shot by the naturalist Diarel. Dr. Heusinger was assured, both by Dr. Besel and by Dr. Fritze, director of the great hospital at Batavia, that all the intermediate gradations between these two animals are well known in Java, and that nobody entertains there the slightest doubt as to the identity of the young red orang and the old black or dark-brown pongo. This, indeed, was the opinion, though only maintained conjecturally, of Tilesius, Cuvier, Lawrence, and Rudolphi. It was supported in the most convincing manner by Mr. Owen, in the first volume of the Transactions of the Zoological Society. Since the publication of that memoir, the identity of the pongo and orang has been called in question by M. Blainville, who has brought forward in opposition to it some observations on the skull of a full-grown orang utang from Sumatra; Borneo having been hitherto the only known habitation of the species. All objections against the identity of the pongo and orang of Borneo turn upon the question whether age is capable of giving rise to such surprising differences of formation as that which display themselves on a comparison of the skulls of the smaller and larger animal; and this is the anatomical, or rather physiological, question which Dr. Heusinger has discussed. He was induced to publish these observations by the expression of Blainville's doubts against an opinion which he had long held as certain. Unfortunately, Dr. Heusinger had never read the excellent memoir of Mr. Owen on the comparative anatomy of the chimpansi and orang utang, of which, as he says, he had only seen an abstract in Müller's Archiv, and a copy of the beautiful pongo skull in the new edition of Prichard's Researches. Had he made himself acquainted with that paper, he would have become more fully aware than he appears to be of the immense importance of attending to varieties in the basis of the skull, as indicative of its most essential characteristics. This remark, which is due entirely to Mr. Owen, is of infinitely greater value than the measurement of the facial angle proposed by Camper, or the comparison of the areas of the cranial span and facial circumference suggested by Cuvier. Heusinger, however, had not sufficient opportunity of instituting this comparison. Of the old pongo he has only a drawing in profile, which is very similar to that given by Owen; but he might have given views of the basis from the skulls in the Würzburg collection. He endeavours to obviate the objections which have been made against Mr. Owen's opinion and his own, by showing that the skull of the young and old animals display a proportionable difference in other species of simiæ, particularly in the cay, the mandril, and the cynocephalus. With regard to the cay, the cebus of Azava, we possess the most accurate information from Reugger, who, in his "Naturgeschichte der Säugthiere von Paraguay," has shown that the young animals of that species undergo most extensive changes in the development of the bony fabric; the skull and the contour of the face being altered at the period when the second dentition takes place, in a manner similar to the changes pointed out by Mr. Owen in the chimpansi and orang. The young and the old mandril differ from each other more widely, both in form and colour, than the orang and the pongo: they were long taken for two distinct species. F. Cuvier and Geoffroy have often observed the transition from one to the other form. Similar variations, depending on age, have been noticed by Ehrenberg* in the cynocephalus hamadryas, who has observed that several species have been constituted by naturalists from the varieties, depending merely on age and sex, which this tribe presents. These remarks suffice to remove all improbability from the opinion maintained by Owen and Heusinger as to the identity of the orang and pongo; and the present work brings some additional evidence in its support, from the positive testimonies of competent observers in the native country of the tribe.

By far the greater part of Dr. Heusinger's memoir is devoted to a discussion of what he calls the fable of the orang utang; by which he means the superstitious veneration paid to apes in different countries. This introduces a learned disquisition on the worship of animals, as practised by the Egyptians, and which, as it is well known, constituted so remarkable a feature in the system of superstitious observances, for which that people are famous through the world. In reference to the worship of the ape in Ethiopia and Egypt, Dr. Heusinger adduces a whimsical conjecture of Ehrenberg's: he thinks that the ancients distinguished but obscurely from each other the lower tribes of mankind and the simiæ. Many races in Abyssinia now wear their hair frizzled out in such a manner as to present a striking resemblance to the cynocephalus hamadryas. It *might* be from a human being, with a similarly frizzled head and a cynocephalic muzzle, born in Ethiopia, perhaps at Merawe, (a place well known to the Egyptians,) that that celebrated people first received the art of writing; for which they gratefully deified the inventor! This may appear very probable to a learned German traveller, who has seen so many wonders in foreign lands; but he must tell us why the inventor of letters, Thoth, is always represented with the head of the ibis instead of that of a monkey.

A chapter in this learned work of Dr. Heusinger's is devoted to the names of the ape in different languages, and to the etymology and affinity of these names; another to the most ancient notices of simiæ in antiquity, which he finds to be in the symbols adopted in the cycle of twelve years, which was very early in use among the astronomers of middle Asia; for an account of which he refers to Abel, Remusat, and Julius Klaproth. Hanamar, the ape of Indian mythology, and the wars of Rana against the monkeys of Lanka-dwipa, are not neglected.

* Symbolæ Physicæ.

